# *Ki67* and *P53* Expression in Relation to Clinicopathological Features in Phyllodes Tumour of the Breast

**DOI:** 10.31557/APJCP.2020.21.9.2653

**Published:** 2020-09

**Authors:** Nurul Atiah Mohd Ali, Ahmad Fazlin Nasaruddin, Syarah Syamimi Mohamed, Wan Faiziah Wan Rahman

**Affiliations:** 1 *Department of Pathology, School of Medical Sciences, Universiti Sains Malaysia, Health Campus, 16150, Kubang Kerian, Kelantan, Malaysia. *; 2 *Department of Pathology, Hospital Sultanah Nur Zahirah, 20400, Kuala Terengganu, Terengganu, Malaysia. *; 3 *Hospital Universiti Sains Malaysia, 16150, Kubang Kerian, Kelantan, Malaysia. *

**Keywords:** Ki67, p53, Phyllodes tumour, breast tumour, immunohistochemistry

## Abstract

**Objective:**

Phyllodes tumour (PT) is a rare fibroepithelial neoplasm of the breast that carries a risk of malignancy. Histopathological examination remains a gold standard for diagnosis. The usage of the immunohistochemical markers of* Ki67* and *p53* acts as a supplement method, particularly for the malignant PT. We aim here to study the expression of these markers in *PT* and to see their relation to the tumour grading.

**Methodology:**

We conducted a retrospective cross-sectional study on 57 archived formalin-fixed paraffin-embedded tissue blocks of PT from the years 2015 to 2018 from two hospitals in East Coast Malaysia. The histopathological examination and immunohistochemical stain for Ki67 and p53 were analysed.

**Results:**

There was an association between clinical descriptive data of skin changes, lump size of more than 3 cm, cytological atypia, stromal hypercellularity, mitosis and immunohistochemistry with the clinical diagnosis of PT. Both marked expression of *Ki67* and* p53* were seen in borderline and malignant PT. Our study showed that in the presence of high mitotic figures, marked expression of *Ki67* was only seen in cases of malignant PT.

**Conclusion:**

We found a significant association of *Ki67* and *p53* expressions, high mitosis and other descriptive histopathological features in malignant PT. Further study with larger sample size is recommended to predict tumour grade and prognosis as well as the disease-free survival of the tumour.

## Introduction

Phyllodes tumour (PT) was initially described as cystosarcoma phyllodes in the year 1838 by Johannes Muller due to its biphasic appearance of benign epithelial elements and fibrosarcomatous stroma (Veneti and Manek, 2001; Lee et al., 2007). This fibroepithelial tumour was characterised by stromal hypercellularity together with cleft like or cystic spaces lined by epithelium.

The incidence of this disease is 1.5% and in a previous study it is said to be very uncommon, with about one in every 100,000 women being affected (Moffat et al., 1995; Testori et al., 2015). Nonetheless, regarding the rarity of this neoplasm, it has a malignant-fatal spectrum (Tan et al., 2016). Asian people in particular tend to have this tumour at a young age with a relatively high rate of recurrence compared to non-Asian people (Teo et al., 2012; Tan et al., 2016).

Similar to fibroadenoma, the neoplastic fibroblast of PT arises from the specialised stroma, in contrast to non-specialised stroma in pseudoangiomatous stromal hyperplasia, with the histogenesis of PT believed to arise de novo, basically from ductal and lobular stroma, involving the interaction between the epithelial and stromal component of the breast tissue. Having an unlimited ability to grow, being classified as stromal overgrowth and being manifested as a large mass that lacks a glandular element, the neoplastic cells in PT are independent of the glandular epithelial, unlike the fibroadenoma. Some studies have stated that hereditary factors do play a part in the development in this tumour, particularly in *TP53* and *MED12* mutation, in a way similar to fibroadenoma, but with additional genetic aberrations of the tumour suppressor genes in PT (Tan et al., 2016).

With the stromal hypercellularity being the key histological feature to differentiate between a PT and fibroadenoma (Lee et al., 2007; Jara-Lazaro et al., 2010), the histological appearance of leafy architecture and stromal cellularity are the gold standard in diagnosing PT (Tan et al., 2016). 

There are overlap features on sonography imaging for both PT and fibroadenoma, as mentioned in many studies, thus necessitating histopathology examination (Chao et al., 2002; Testori et al., 2015). With the pathologists still having difficulty in distinguishing between benign PT and the cellular fibroadenoma as both carry similar microscopic features, except for the presence of minimal stromal cellularity in the diagnosis of the former (Tan et al., 2016), some of the studies have said that good fine needle aspiration composed of both epithelial and stromal component can yield a better differentiation between PT and fibroadenoma (Veneti and Manek, 2001; El Hag et al., 2010). 

With the evidence of necrosis and heterologous elements, an index of suspicion arises of malignant PT, although it is not counted as a criterion for diagnosis (Moffat et al., 1995). According to the World Health Organisation (WHO), PT can be classified into three major categories: benign; borderline; and malignant based on five major histological features, which are degree of stromal cellular atypia, stromal hypercellularity, stromal overgrowth, mitotic count and infiltrative or pushing margin. According to WHO, PT being comprised of a group of fibroepithelial neoplasms that resembles fibroadenoma but with other distinctive histological features. Their sarcomatous element may also resemble the pure stromal sarcoma. 

The recurrence of tumour is defined as a similar histological type tumour that presents at a similar quadrant. However, tumours that arise in a different quadrant or from the contralateral breast are known to be new tumours. Positive margin or incomplete surgical excision contribute to this tumour recurrence (Teo, Cheong and Wong, 2012). Another study showed that high mitosis is also associated with the risk of recurrence, and in terms of metastasis capability, a similar study showed that it is contributed to by the presence of stromal hypercellularity but not by the existence of stromal overgrowth, cytological atypia, tumour necrosis and a heterologous component (Moffat et al., 1995).

The usage of immunohistochemistry (IHC) in diagnosing a benign PT is not pronounced as compared to the other tumour except in a case of malignant PT (Korcheva et al., 2011). Multiple studies done to identify histological parameters that predict behaviour have yielded variable results. 

TP53 is a ubiquitous protein in the human body and a well-known potent tumour suppressor gene. Therefore, any upregulation expression of this protein can be detected by IHC method. For many years, the role of TP53 as a tumour marker in many other tumours has been extensively studied, including in PT; however, the utility of this protein as a prognostic biomarker has remained uncertain (Niezabitowski et al., 2001; Aydoğanet al., 2016).

Ki67 is a non-histone nuclear protein that is strongly correlated with the mitotic index of the tumour; hence it is an important proliferative marker used in IHC study (Shoker et al., 2001), and peaks during the G2M phase of the cell cycle (Song and Yoon, 2008). Some studies have showed that it can also be used as a predictive marker as it measures the probability of recurrence, particularly in malignant PT (Niezabitowski et al., 2001; Tseet al., 2010). A similar study shows that both the IHC marker of Ki67 and p53, with evidence of mitotic activity, play an individual role in prognostication elements. 

Approximately 3% of normal breast epithelial cells show positivity for Ki67 staining. It will be higher in the invasive breast carcinoma, particularly in hormonal negative tumours (Shoker et al., 2001). The expression of *Ki67* staining in malignant PT fluctuates from 15%–100%, whilst it is less than 25% in a case of benign PT (Tse, Niu and Shi, 2010). In the case of malignant PT with the presence of osteosarcomatous differentiation; exhibits higher Ki67 index. However, a similar study showed that, in cases of morphologically benign PT, more than 10% staining of Ki67 displayed is a risk for malignant changes (Ribeiro-Silva et al., 2006). On top of it, other markers that can be used for PT are CD117 (ckit), ER, PR, CD 31, CD10 (CALLA), and EGFR (Niezabitowski et al., 2001; Shoker et al., 2001; Lee, 2008; Song and Yoon, 2008; Jara-Lazaro et al., 2010; Korcheva et al., 2011; Noronha et al., 2011).

The mainstay of treatment remains the surgical procedure (El Hag et al., 2010; Mishra et al., 2013), with the choice of procedure depending on the classification of this tumour; however, the breast-conserving therapy is most preferable. In benign phyllodes, tumour excision with additional 1 cm margin of normal breast tissue suffices. The risk of local recurrence is as high as 27% in the case of malignant PT (Teo et al., 2012), and it has a possibility of metastasis as well as fatal risk. 

*Research question*

As aforementioned, the histopathological appearance is considered the gold standard in diagnosing the breast neoplasm of PT. Our research is aimed to determine the expression tumour related markers (*Ki67* and *p53*) based on grading using IHC test. This non-invasive markers test is hopefully capable of assisting in the diagnosis of tumours, particularly for malignant PT types, ahead of time. 

## Materials and Methods

A retrospective cross-sectional study was conducted in the Hospital Universiti Sains Malaysia (USM) and the Hospital Sultanah Nur Zahirah (SNZ), Kuala Terengganu, for patients diagnosed from 1st January 2015 until 31st December 2018. The study was approved by the Human Ethics Committee of the School of Medical Sciences, the Universiti Sains Malaysia (USM/JEPem/18030185) and the Medical Research and Ethics Committee Ministry of Health, Malaysia (NMRR-19-2255-49034). Patients’ clinicopathological data were retrieved from the medical record unit and laboratory information system. A proforma form was used for data collections.

A total of 57 slides of haematoxylin and eosin (H &E) and formalin fixed paraffin embedded (FFPE) blocks were retrieved. The previous H&E stained slides were reassessed for histopathological examination (HPE) scoring. Subsequently, the FFPE blocks were further stained with immunohistochemistry (IHC) study of Ki67 and p53. Tables were used for the HPE and IHC scoring of the tumour tissue.

Ki67 and p53 are a common IHC at the laboratory, however, it is not routinely done in PT diagnosis. Using a DAKO technology, Monoclonal Mouse Anti-Human p53 Protein clone DO-7 and Monoclonal Mouse Anti-Human Ki-67 Antigen Clone MIB-1 were used as a primary antibody. For both p53 and Ki67 staining, the primer antibody was diluted at a ratio of 1:100, while the secondary antibody used was horseradish peroxidase (HRP) polymer solution, using a DAKO-real HRP RABBIT-MOUSE (ENV). The internal control for both Ki67 and p53 was use of lymphocytes and normal colon tissue.

**Figures 1:**
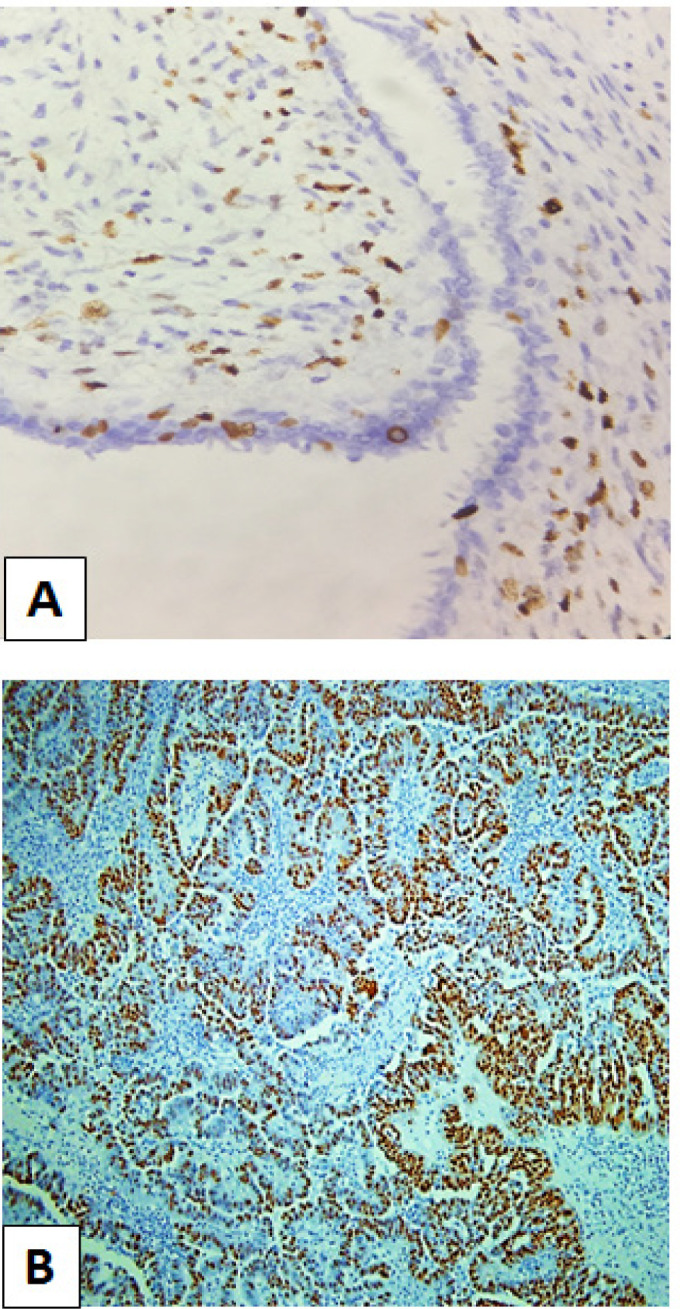
(A) Marked expression of *Ki67* as defined by more than 30% of nuclear staining (400x magnification). (B) Marked expression of *p53* as defined by more than 80% of nuclear staining (200x magnification)

Semiquantitative scoring analysis was used by two observers. The presence of nuclear staining for both Ki67 and p53 was considered as positive. Based on the study by Vani et al., (2016), the measurement of Ki67 is defined as any detectable brown staining of the nucleus. It is categorised into three aspects: Mild (0%–10%); Moderate (11%–30%); and Marked (more than 30%). Marked expression as depicted in Figure 1(A). Whereas for the p53 protein expression, based on the studies by Niezabitowski et al., (2001). the assessment would be graded into three similar categories: Mild (0–50%); Moderate (51–80%); and Marked (more than 80%). The example of marked expression is displayed in Figure 1(B). 

The data was analysed using a software IBM SPSS version 24. The association between clinicopathological data and protein expression of Ki67 and p53 were analysed using Pearson Chi Square test and a level of significance (p-value) was set at <0.05.

## Results

A total number of 57 tissue samples were recruited for this study based on patients diagnosed with PT at Hospital USM and Hospital SNZ. The clinicopathological data are presented in Table 1. The majority of the samples were from Malay (98.2%), except for one for Chinese (1.8%). Data analysis showed that for the majority of patients the age group was more than 20 years (n = 54, 94.7%). All of them presented with a palpable lump, in which the size of less than 3 cm was solely attributed to benign PT. All patients with malignant PT showed a lump size of more than 3 cm (n = 5, 16.7%). About 17.5% (n = 10) of the patients presented with skin changes, the majority seen in malignant PT, whilst two of them were seen in both benign and borderline PT. 

From the histopathological aspect, more than half of the patients showed no evidence of cellular atypia 68.4% (n = 39) and presence of stromal overgrowth (n = 44, 77.2%), as compared to the stromal hypercellularity (n = 27, 47.4%). In many cases, mitosis of less than 5 (n = 44, 77.2%) was shown. Based on Table 1, the proportion of benign PT was 73.7%. In the majority of *Ki67* (89.5%) and *p53* (59.5%) cases, protein expression was graded in the mild group. 

Table 2 shows the association between the clinicopathological data with the diagnosis. There were significant associations between clinical descriptive data (skin changes [p = 0.004]), histopathological features (atypia status [p < 0.001], stromal hypercellularity [p = 0.012]) and mitosis (p < 0.001) with the diagnosis. 

Pearson Chi Square test was used to examine the association between the clinicopathological data and protein expression of* Ki67* and P53 in phyllodes tumour. Based on Table 3, there was a significant association between the distribution group of Ki67 and p53 in relation to the diagnosis (both p < 0.001). It was shown that most of the negative/mild Ki67 and p53 protein expression fell into the benign group 42(82.4%) and 32(94.1%), while marked expression of both *Ki67* and* p53* fell into the malignant group 4(100%) and 3(50%). 

In case of the borderline PT, our study showed a marked staining for p53 marker 3 (50%) only and negative/mild staining for Ki67 9(17.6%). Meanwhile, for the malignant PT it shows the presence of marked expression for the dual immunohistochemistry marker of *Ki67 *(100%) and* p53* (50%). Therefore, Ki67 protein expression is beneficial for the case of malignant PT, on top of other histopathological and clinical suspicions. 

In our study, relevant important findings of skin changes, lump size of more than 3cm and evidence of cytological atypia together with the presence of marked p53 protein expression shall raise the suspicion of at least borderline PT. We can conclude that the presence of very high mitosis and marked protein expression of Ki67 are seen in cases of malignant PT. 

**Table 1:** Clinicopathological Data of the Case Study (n=57)

Variables	N (%)
Age	
Less than 20	3 (5.3)
More than 20	54 (94.7)
Race	
Malay	56 (98.2)
Others	1 (1.8)
Skin changes	
No	47 (82.5)
Yes	10 (17.5)
Lump size	
Less than 3 cm	27 (47.4)
More than 3 cm	30 (52.6)
Treatment	
Defaulted	7 (12.3)
Lumpectomy	29 (50.9)
Mastectomy	12 (21.1)
WLE	9 (15.8)
Atypia	
Yes	18 (31.6)
No	39 (68.4)
Stromal hypercellularity	
No	30 (52.6)
Yes	27 (47.4)
Stromal overgrowth	
No	13 (22.8)
Yes	44 (77.2)
Mitosis	
<5	44 (77.2)
5-9	7 (12.3)
>9	5 (8.8)
Phyllodes tumour	
Benign	42 (73.7)
Borderline	9 (15.8)
Malignant	6 (10.5)
Ki67	
Negative/mild	51 (89.5)
Mod	2 (3.5)
Mark	4 (7.0)
p53	
Negative/mild	34 (59.5)
Mod	17 (29.8)
Mark	6 (10.5)

**Table 2:** Clinicopathological Data in Relation to the Diagnosis (n=57)

Variables	Diagnosis			P value
	Benign	Borderline	Malignant	
	n (%)	n (%)	n (%)	
Clinical Data				
Age				>0.950
Less than 20	3 (100.0)	0 (0.0)	0 (0.0)	
More than 20	39 (72.2)	9 (16.7)	6 (11.1)	
Race				>0.950
Malay	41 (73.2)	9 (16.1)	6 (10.7)	
Chinese	1 (00.0)	0 (0.0)	0 (0.0)	
Treatment				<0.001
Defaulted	6 (85.7)	1 (14.3)	0 (0.0)	
Lumpectomy	27 (93.1)	2 (6.9)	0 (0.0)	
Mastectomy	3 (25.0)	4 (33.3)	5 (41.7)	
WLE	6 (66.7)	2 (22.2)	1 (11.1)	
Skin changes				0.004
No	38 (80.9)	7 (14.9)	2 (4.3)	
Yes	4 (40.0)	2 (20.0)	4 (40.0)	
Lump size				0.052
Less 3 cm	24 (88.9)	2 (7.4)	1 (3.7)	
More 3 cm	18 (60.0)	7 (23.3)	5 (16.7)	
Histopathological examination (HPE)				
Atypia				<0.001
Yes	4 (22.2)	8 (44.4)	6 (33.3)	
No	38 (97.4)	1 (2.6)	0 (0.0)	
Stromal hypercellularity				0.012
No	26 (86.7)	4 (13.3)	0 (0.0)	
Yes	16 (59.3)	5 (18.5)	6 (22.2)	
Stromal overgrowth				0.33
No	12 (92.3)	1 (7.7)	0 (0.0)	
Yes	30 (68.2)	8 (18.2)	6 (13.6)	
Mitosis				<0.001
<5	42 (93.3)	3 (6.7)	0 (0.0)	
5-9	0 (0.0)	6 (85.7)	1 (14.3)	
>9	0 (0.0)	0 (0.0)	5 (100.0)	
Immunohistochemistry (IHC)				
Ki67				<0.001
Negative/mild	42 (82.4)	9 (17.6)	0 (0.0)	
Mod	0 (0.0)	0 (0.0)	2 (100.0)	
Mark	0 (0.0)	0 (0.0)	4 (100.0)	
p53				<0.001
Negative/mild	32 (94.1)	2 (5.9)	0 (0.0)	
Mod	10 (58.8)	4 (23.5)	3 (17.6)	
Mark	0 (0.0)	3 (50.0)	3 (50.0)	

**Table 3:** Distribution of Immunohistochemistry (IHC) Results Based on Diagnosis

Variables	Diagnosis			P value^a^
	Benign	Borderline	Malignant	
	n (%)	n (%)	n (%)	
Ki-67				<0.001
Negative/Mild	42 (82.4)	9 (17.6)	0 (0.0)	
Mod	0 (0.0)	0 (0.0)	2 (100.0)	
Mark	0 (0.0)	0 (0.0)	4 (100.0)	
p53				<0.001
Negative/Mild	32 (94.1)	2 (5.9)	0 (0.0)	
Mod	10 (58.8)	4 (23.5)	3 (17.6)	
Mark	0 (0.0)	3 (50.0)	3 (50.0)	

^a^, Pearson Chi Square test

## Discussion

PT is defined as a biphasic tumour of epithelial component within a neoplastic spindle cell stroma, and due to its intratumoural heterogeneity, the features might be intermingled, thus, in the presence of suspicious foci of heterologous components, the diagnosis should be upgraded (Zhang and Kleer, 2016). Based on the WHO classification, it is categorised into benign, borderline and malignant, distinctive by their histological appearances.

We find that in a case of benign PT, patients usually presented with an innocuous small breast lump. In our study, larger tumour size of more than 3 cm and presence of skin changes did not correlate with the histological findings, and in the case of larger tumour size and skin ulceration, the malignant tumour is prime because most of them are usually manifested late in their disease course. In the presence of lymph node; in which it is rare to be positive nodes in benignity disease, therefore it prompts malignant PT (Testori et al., 2015). However, this is not questioned in our study and might be investigated in later studies.

On top of its ultrasonographic features, needle biopsy is found to be superior to cytological analysis in order to differentiate PT from a more common differential diagnosis of cellular fibroadenoma, as both show the presence of larger stroma fragments and numerous stromal bare nuclei (Veneti and Manek, 2001; Chao et al., 2002; El Hag et al., 2010; Berner and Sauer, 2011; Tsang et al., 2011; Tan et al., 2016). 

The prime key histological features will be a stromal hypercellularity that is found immediately beneath the subepithelial region (Jara-Lazaro et al., 2010; Tsang et al., 2011). Hence, this appearance supports the hypothesis that this PT arises from the periductal areas instead of from intralobular stroma cells (Kleer et al., 2001; Tse, Niu and Shi, 2010).

Based on our study, where other microscopic findings of cytological atypia, permeative margins, stromal hypercellularity and stromal overgrowth are compulsory in making the diagnosis but not for tumour grading, amongst the five key histological features mitosis plays a vital role to aid in the diagnosis of malignant PT, in which this diagnosis shall be considered in the presence of mitosis of more than 9. Having said that, the presence of the above features increases the risk of local recurrence and metastasis (Chao et al., 2002).

Histologically, to diagnose malignant PT, the differential of sarcoma should be lingered as it shows a stromal differentiation of fibrosarcomatous type. Both are having negative expression of basal keratin and beta catenin, so the expression of *Ki67* index together with stromal positivity of *CD34* may aid in the diagnosis of PT over the sarcoma (Lee, 2008). Other than that, genetic study is also helpful since this PT has a lack of oncogenic mutation as compared to other carcinomas, sarcomas and melanomas (Korcheva et al., 2011). 

With the risk of local recurrence also being higher with higher grade, some studies conclude that the size does not correlate with local recurrence except for the stromal overgrowth (Aydoğan, Tasçi and Sagara, 2016). Therefore, bringing along the risk of local recurrence and metastatic property, proper pre-operative diagnosis and post-operative management are crucial. (Mishra et al., 2013; Lu et al., 2019). 

With lymph node resection not being recommended initially and the need for further adjuvant relying on subsequent follow-up of histopathological examination (Chen et al., 2005; Telli et al., 2007), a wide local excision with 1 cm of free margin acts as a primary treatment intraoperatively. However, in our study we evaluated neither the risk of local recurrence nor the metastatic capability. Other than that, the tumour behaviour was also predicted by surgical-margin status (Esposito et al., 2006).

In recent years, there have been extensive studies of evaluating the usage of immunohistochemistry (IHC) markers to aid in the diagnosis of PT. With the usual markers commonly used being the proliferative marker of Ki67 and a tumour suppressor gene of p53, other markers have been hormonal receptor, an angiogenesis group of marker (i.e. CD31), epidermal growth factor, CD117, CD10, pH3, Actin, BCL2 and Cyclin D1 (Kleer et al., 2001; Niezabitowski et al., 2001; Shoker et al., 2001; Esposito et al., 2006; Lee, 2008; Song and Yoon, 2008; Bose et al., 2010; Tse, Niu and Shi, 2010; Jara-Lazaro et al., 2010; Korcheva et al., 2011; Noronha et al., 2011; Wang et al., 2017).

The p53 protein of tumour suppressor gene is the initial marker that was studied as it is extensively altered in breast cancer as well as in other neoplastic tumours (Tse et al., 2010), and is found to be ubiquitous in our body, readily functioning as a regulator in cell cycle, cell differentiation and DNA cell repair. In our study, the expression of this marker presence in all cases of PT with different intensity. The benign cases are expressed up to moderate intensity only, whereas for malignant *PT* the expression is equal between moderate and marked staining. As concluded by our study, the marked expression of *p53* is seen equally in both cases of borderline and malignant PT.

Based on our study, the malignant PT shows moderate to marked intensity for Ki67 staining, and this cell cycle proliferative marker is a well-known immunohistochemical protein that has been helpful in distinguishing many types of actively proliferating tumour masses in our body parts. 

However, in a case of benign and borderline tumour, this Ki67 is expressed in less than 10% of the nuclear staining, thus falling into the negative/mild group. We also found that there is significant correlation between the Ki67 index and tumour histological type, particularly of high mitosis (more than 9/10 HPF), on top of other microscopic features. 

Although neither* p53* nor *Ki67* expression are reliable to predict the rate of recurrence of PT (Kleer et al., 2001; Niezabitowski et al., 2001), our study showed that *Ki67 *expression is helpful in identifying difficult cases of malignant PT. The combination with p53 positivity acts as an added value.

In many researches, it was found that this Ki67 index plays an important prognostic factor in PT. However, in one study emphasising that the usage of these biomarkers does not significantly improve the prognostic outcome, they emphasised that the histological features remained a predictive factor of the biological behaviour of the PT (Tan et al., 2016).

Tumour cells of higher grade will express a significant staining of p53 and CD117 protein. However, the expression of *Cyclin D1* is not associated with the tumour grading (Korcheva et al., 2011). Similar to another study, this study also suggests that the combination of p53, Ki67 and CD117 protein expression is helpful in distinguishing the tumour grade (Noronha et al., 2011; Tse, Niu and Shi, 2010). Having said that, the expression of* Cyclin D1* is non-conclusive since it also expresses in normal and cancerous breast tissue (Shoker et al., 2001; Noronha et al., 2011).

Therefore, we suggest dividing this Ki67 index in further research into low and high grade of a more precise group, as it may be helpful in predicting the prognosis as well as the disease-free survival of the tumour. To date, the clinical use of molecular genetics is still under investigation. Few studies involving the PDGFRA and the KIT mutation have showed no pathogenetic role in breast PT (Bose et al., 2010; Korcheva et al., 2011). 
